# The utility of physical activity questionnaires among African origin populations; lost in translation?

**DOI:** 10.21203/rs.3.rs-5529358/v1

**Published:** 2024-12-12

**Authors:** Jessica C. Davies, Candice Choo-Kang, Larske Soepnel, Hayli Geffen, Asanda Mtintsilana, Chad Africa, Pascal Bovet, Bharathi Viswanathan, Kweku Bedu-Addo, Prince Oti Boateng, Kingsley Apusiga, Oscar Akunor Dei, Terrence E. Forrester, Estelle V. Lambert, Nandipha Sinyanya, Brian T. Layden, Jack A. Gilbert, Gertrude Ecklu-Mensah, Cara Joyce, Amy Luke, Lara R. Dugas

**Affiliations:** University of Cape Town; Loyola University Chicago; University of Cape Town; University of Cape Town; University of Cape Town; University of Cape Town; Centre universitaire de médecine générale et santé publique, Lausanne; Ministry of Health; Kwame Nkrumah University of Science and Technology; Kwame Nkrumah University of Science and Technology; Kwame Nkrumah University of Science and Technology; Kwame Nkrumah University of Science and Technology; University of the West Indies; University of Cape Town; University of Cape Town; University of Illinois at Chicago; University of California San Diego; University of California San Diego; Loyola University Chicago; Loyola University Chicago; University of Cape Town

**Keywords:** Self-reported physical activity, objectively measured physical activity, non-communicable diseases, obesity

## Abstract

**Background:**

Physical activity (PA) is an important preventive factor of non-communicable diseases (NCDs), particularly cardiovascular disease, yet progress towards reducing physical inactivity in populations is slow. Population-levels of PA are most often estimated using self-report questionnaires in population surveys, such as the Global PA Questionnaire (GPAQ), which may not accurately reflect objectively measured PA, such as accelerometers. The aim of the current study was to compare self-report vs objectively measured PA across 5 African-origin populations.

**Methods:**

Approximately 2,000 African-origin men and women (35–55 yrs) were enrolled in a prospective cohort study to explore the relationship between lifestyle and cardiometabolic health. Participants were from Ghana, South Africa (SA), Jamaica, Seychelles, and the United States (US). Data collection included objective PA (accelerometer) and self-reported PA (GPAQ). WHO defines “sufficiently active” as performing >150 minutes of moderate intensity PA per week, or >600 MET*min/week (metabolic equivalent of task in minutes/week). Logistic regression was used to determine the proportion of participants that were physically active by accelerometry, among those who were found as sufficiently active based on GPAQ.

**Results:**

1,161 participants had complete self-reported PA and accelerometery data. Overall, 23.5% were classified as sufficiently active by both PA measures, while 38.2% of those classified as sufficiently active based on self-reported PA did not meet the criteria using objective monitoring (sensitivity of GPAQ: 74%, specificity: 44%). Among participants who were classified as sufficiently active according to the questionnaire (n=717), participants from Ghana (OR=3.2, p<0.01), SA (OR=5.3, p<0.01), Jamaica (OR=2.3, p=0.01), and Seychelles (OR=1.9, p=0.03) were more likely to be similarly classified as sufficiently active based on accelerometry, compared to those from the US, and men (OR=2.9, p<0.01) more likely than women. Finally, obese participants had 0.4 times lower adjusted odds to be similarly classified as sufficiently active using objective measures compared to non-obese participants (p<0.01).

**Conclusion:**

Our findings further underscore difficulties in interpreting self-reported PA, which may furthermore vary across different settings and socio-economic settings. Given the importance of PA interventions for reducing the NCD burden, future research should explore tailored approaches to better understand self-report PA accuracy and to better assess the dose-response between PA and cardiometabolic risk.

## Introduction

Annually, 41 million deaths are attributable to non-communicable diseases (NCDs), including cardiovascular disease (CVD) [1]. More than 75% of these deaths occur in low- and middle-income countries, consistent with the much larger population size living in these countries than high income countries. Physical inactivity contributes significantly to the occurrence of NCDs [2–12]; 7.2% and 7.6% of all-cause and CVD deaths, respectively, are attributable to physical inactivity [6]. In the *Global action plan on physical activity 2018–2030 (GAPPA)*, the World Health Organization (WHO) set a target of 15% reduction in the global prevalence of physical inactivity in adults and in adolescents by 2030 [8]. However, progress towards this target has been slow, unequal, and has been negatively impacted by the Covid-19 pandemic [4]. This reinforces the urgency and importance with which evidence-based, effective policies as outlined in GAPPA should be implemented globally to reduce the financial and systemic burden of NCDs on health systems [4].

In 2020, the WHO published revised *Guidelines on Physical Activity and Sedentary Behaviour* [13], which recommend that all adults aged 18–64 should be, throughout the week, engaging in at least 150–300 minutes of moderate-intensity aerobic PA (MPA), or at least 75–150 minutes of vigorous-intensity aerobic PA (VPA), or an equivalent combination of moderate- and vigorous-intensity PA (MVPA). Regular muscle-strengthening activities are recommended for all age groups. Reducing sedentary behaviour was also recommended across all age groups and abilities [13, 14]. The WHO developed the Global Physical Activity Questionnaire (GPAQ), to determine, through self-report, whether participants are meeting these WHO recommendations for PA across work-related activities, travel, and recreational activities [15]. In 2022, an estimated 81% of adolescents and 27.5% of adults do not meet these recommendations [4]. Worryingly, great differences are seen in PA levels according to regions, countries, age groups and sex, as well as socioeconomic status, disability and pregnancy [4, 8]. The global implementation of the WHO PA targets, the significance of PA for health, and the discrepancies in PA across population groups emphasize the public health importance of accurately and reliably measuring PA in various global settings.

Self-reported measures of PA are widely used in research and healthcare settings, and while questionnaires such as GPAQ are simple to administer, and provide insight into the domains or context in which physical activity is being undertaken [12, 15–17], self-reported PA is well-known to potentially lead to errors in determining the total volume and intensity of PA based on device-measured estimates [16, 18–20]. For example, PA collected using questionnaires has consistently been shown to generally overestimate a person’s PA compared to PA measured with accelerometer [16, 21–26], and this bias may vary according to numerous factors such as age, sex, literacy, memory, obesity or characteristics of the interviewers when questionnaire are administered face-to-face by health officers [27, 28]. This may result from the structure of some questionnaires (e.g., GPAQ) requiring participants to estimate PA within bouts of PA lasting at least 10 minutes consecutively (so that PA on smaller time periods, although very frequent in daily life of most individuals, are not included), leading to rounding up of time spent performing PA, and because questionnaires ask participants to report domain-based PA levels (i.e., at work -both for paid work and activities at home-, at leisure time and walking time not included in PA reported at work or for leisure time). Additionally, previous studies have found that many individuals tend to overestimate their actual PA and other healthy behaviours due to social desirability of portraying oneself as partaking in healthy practices [12, 29]. Due to varying living environments, disease burden, health literacy, and socioeconomic factors, this bias may therefore shift as countries undergo the epidemiologic transition; a changing pattern in population age distributions, mortality, fertility, life expectancy, and causes of death [30, 31]. Tucker et al. [24] found that as many as 59.6% of the US population met the PA guidelines through self-report, but that in fact only 8.2% of the same populationmet the PA guidelines when considering objectively measured PA data. In an international systematic review conducted by Prince et al, self-report measures were found to both under- and overestimate PA when compared to device-measured estimates, highlighting the issues surrounding reliance on self-report measures, and the need for valid, accurate and reliable measures of PA [12].

Alternatively, PA data can be collected using accelerometers, which are able to identify accelerations of a body through motion detectors [16]. This results in unbiased, practical, and reliable PA estimates, particularly for land-based and ambulatory PA. However, accelerometers are expensive, require trained staff and detailed analyses [16, 18], and are not able to record certain activities, such as bicycling, swimming, and household chores, activities with added weights, or a change in gradient of the workout surface [16]. They also fail to capture context or the domain of physical activity. Yet, newer technologies such as PA measurement provided in mobile phones, watches and other portable devices, which deliver objective data in inexpensive and user-friendly manners for all individuals, are likely to offer alternative means, including in resources constrained settings.

A better understanding of PA estimates by self-report compared to accelerometery in population groups at various stages of the epidemiologic transition, for whom scarce data is available, is useful for interpreting PA data, establishing how PA data relates to health risks, and translating this to appropriate recommendations. In this paper, we aimed to compare self-reported and objectively measured PA across 5 African-origin populations, by determining the extent to which participants were classified as meeting PA guidelines by both methods. Secondly, among those who were characterised as being sufficiently active by self-report (GPAQ), we aimed to identify participant characteristics associated with being sufficiently active by the gold standard PA measure, accelerometry. This subset was chosen as this allowed us to identify characteristics of the individuals who may or may not be overestimating PA by self-report.

## Methodology

### Study design and settings

This is a cross-sectional study using data from the Modeling the Epidemiologic Transition Study (METS)-Microbiome cohort study. The METS-Microbiome study [32] is a follow-up study of METS, a prospective, well-established cohort study [33], with sites in five African-origin populations (Ghana, South Africa, Jamaica, Seychelles, and the US). These five sites represent the epidemiologic transition spectrum, based on their Human Development Index (HDI), with Ghana and the US on each end of the spectrum [32]. Detailed protocols of METS and METS-Microbiome have previously been published [32, 33].

### Recruitment and enrolment

METS-Microbiome enrolled approximately two thousand adults (n=2,000, approximately 400 from each of the five study sites) between the ages of 35 and 55 years between January 2017 and December 2019. In the current retrospective analysis, only participants with complete data for both self-reported and accelerometery-measured physical activity from METS-Microbiome were included.

The protocol for METS-Microbiome was approved by the Institutional Review Board of Loyola University Chicago, IL, US; the Committee on Human Research Publication and Ethics of Kwame Nkrumah University of Science and Technology, Kumasi, Ghana; the Research Ethics Committee of the University of Cape Town, South Africa; the Board for Ethics and Clinical Research of the University of Lausanne, Switzerland; the Heath Research Ethics Committee of the Ministry of Health of Seychelles; the Ethics Committee of the University of the West Indies, Kingston, Jamaica; and the Health Sciences Institutional Review Board of the University of Wisconsin, Madison, WI, US. All participants provided written informed consent prior to enrolment in the study [32–34].

### Research procedures and data collection methods

At each site, all measurements were completed at outpatient research clinics in the early morning following an overnight fast [33, 35].

### Physical activity measurements

#### Objective PA measurement by accelerometry

The Actical accelerometer (Phillips Respironics, Bend, OR, US) was used to objectively measure PA, as previously described [34, 35]. The accelerometer was worn just behind the right hip at the level of the waist, for an 8-day period (i.e., 6 full days with two partial days on either end of the period). The original METS study determined a good level of reliability with an interclass correlation coefficient of 0.83–0.92 across the five sites [33]. The study participants were requested to always wear the monitors, including while sleeping but excluding while immersed in water (i.e., while bathing, showering or swimming) [34]. Due to there being no global guidelines surrounding the definition of sleep-time vs. awake-time for accelerometry data which is collected for a 24-hour period, the time period for assessing PA conducted daily was between 07:00 and 23:00, in order to standardise measurements [35].

Accelerometry data management and analysis has been described in full previously [33–35]. Briefly, a SAS macro programme was used to summarise the raw accelerometer data to determine non-wear time from ≥90 minutes of continuous zero activity counts [33], based on visual inspection of wear/non-wear patterns [35]. For a days’ entry to be valid, the measurement period should be at least 10 hours of wear time, i.e., wearing the accelerometer for ≥62% of available wear time, and participants were excluded from analysis if they had less than four valid days available [34].

“Activity counts” are derived from raw accelerometer data, and they correspond with the frequency and magnitude of acceleration, for further analysis, enabling published cut-points to be used to asses sedentary behaviour, light-, moderate- and vigorous-intensity PA as follows: sedentary behaviour <100 counts per minute (cpm), light-intensity PA: 101–1534 cpm, moderate-intensity PA 1535–3959 cpm and vigorous-intensity PA ≥3960 cpm [36, 37]. Minutes were defined according to the National Center for Health Statistics protocol for analysis of accelerometry data in the National Health and Nutrition Examination Survey (NHANES) [33]. This may be presented as the overall time in minutes combined in intervals of either 1- or 10-minutes [35]. Data are presented as total activity counts divided by total wear time as an overall measure of average PA intensity, and average counts and time 1-minute bouts of moderate-to-vigorous activity (MVPA) [35].

Using accelerometery data, participants were classified as sufficiently physically active if they met the WHO recommendation of at least 150 minutes per week of moderate-to-vigorous intensity PA [34, 38].

#### Self-reported PA measurement by GPAQ

Self-reported PA was assessed using the GPAQ (version 2) [39], which was administered by centrally trained staff. This included determining the number of days per week the participant reported being active across three domains: travel, occupation, and leisure or recreational, in minutes per day. PA was probed by asking participants to recall their PA in the different domains if they accumulated more than 10 minutes in a single bout. In accordance with the GPAQ analysis guide, moderate and vigorous occupational; moderate-to-vigorous recreational activities; moderate activities for travel purposes (i.e., walking, bicycle riding); and sedentary activities were determined. Subsequently, the daily average number of minutes spent performing different PA activities was calculated. Participants were classified as sufficiently active according to GPAQ if they met the equivalent of the WHO recommendation at 600 MET*min/week [34, 38].

### Clinical measures and health history

Weight and height measurements were performed once the participants were barefoot and wearing minimal light clothing. Weight (in kilograms - kg) was recorded to the nearest 0.1kg, and height (in centimetres - cm) was recorded to the nearest 0.1cm. Body mass index (BMI) was calculated as kg/m^2^ [34]. Obesity was defined as being a BMI ≥ 30kg/m^2^ [40]. Fasting capillary glucose levels were determined. Participants with a blood glucose level ≥140 mg/dl in Ghana and ≥125 mg/dl in all other sites were defined as diabetic [41]. Systolic and diastolic blood pressure and pulse were measured using the Omron Automatic Digital Blood Pressure Monitor (model HEM-747Ic, Omron Healthcare, Bannockburn, IL, USA). With the antecubital fossa at heart level, three measurements were made at each of two time points separated by approximately 60 minutes. Participants were classified as hypertensive with a mean systolic and diastolic blood pressure ≥130/80 mmHg or on current treatment.

A basic demographic, health and socio-economic history was obtained from the participants by interviewer-administered questionnaires. An occupation questionnaire from the U.K. National Statistics Socio-economic Classification (NS-SEC), 2000 edition was used to determine, in more detail, individual occupation [35]. Smoking status was determined through questions asked relating to the use of cigarettes, cigar or pipe smoking, and chewing tobacco use.

### Data analysis

Descriptive statistics were completed to summarise the participant characteristics at each of the study sites, through medians and interquartile ranges (IQR) for continuous measures and proportions for categorical variables (Table 1). The results were stratified according to site and sex.

Sensitivity and specificity analyses of GPAQ, compared to accelerometery as the gold standard, were conducted on the entire cohort, as well as stratified by obesity status. Deming regression and Lin’s Concordance Correlation Coefficient was used to measure the association between GPAQ and accelerometry. Additionally, Bland Altman plots were used to visualise and quantify the agreement between the two PA measures.

To address the second aim, among participants who were categorised by self-report as being sufficiently activity, we identified characteristics associated with also being sufficiently active by Actical. Using the subset of participants who met PA guidelines according to GPAQ (n = 717), we performed multivariable logistic regression. The outcome considered was meeting PA guidelines by accelerometry (yes vs. no). The variables included in the model were site, sex, age, obese, work, manual labour, smoking, diabetes, and hypertension. Covariates were included based on *a priori* hypotheses.

## Results

### Demographic and health related findings

The descriptive characteristics of the 1,161 participants (Supplementary Figure 1) included in this study can be seen in Table 1. The median age for the cohort was 43 years (IQR: 37, 49). Median BMI differed substantially between the sites, with the South African men having the lowest (21.4; IQR: 19.6, 23.6) and the US women having the highest BMI (35; IQR: 29, 40). For both men and women, measures of obesity tended to align with the countries’ HDI ranking, with higher ranked countries experiencing higher levels of obesity compared to lower ranked countries (Table 1), with the highest obesity prevalence in the US participants (34% and 70%, respectively for men and women).

Among men, the Ghanaian participants had the highest employment rate (98%), and the highest manual labour rate (72%), while the South African men had the lowest employment rate (54%) and manual labour rate (23%). The Ghanaian men had the lowest prevalence of smokers (1.8%), diabetes (0%), and hypertension (32%). The South African men smoked the most (79%), while the Seychellois men had the most diabetes (23%), and the US participants had the most hypertension (76%).

Among women, the highest work rate was found in the Seychellois women (96%), while the lowest was found in the South African women (29%). The proportion of women performing manual labour was lowest among the Seychellois participants (16%), and highest among the Ghanaian participants (43%). Similarly to results among the male participants, the lowest hypertension prevalence (25%) was found in the Ghanaian women. None of the Ghanaian women smoked, with the South African women having the highest smoking rate (20%).

### Physical activity parameters

PA results as measured by accelerometery and GPAQ are presented in Table 2. Amongst both men and women, the South Africans have the largest proportion of participants who are sufficiently active as measured by accelerometery (78% of the men and 24% of the women), while the US have the smallest proportion (30% of the men and 6.8% of the women). Self-reporting sufficient PA according to GPAQ was highest among the Ghanaian participants (86% of men and 72% women), and lowest among the Seychellois men (55%) and Jamaican women (44%).

### Comparing self-report to objectively measured PA

Of the true positive (i.e., being sufficiently active by accelerometery), 74% (69% – 79%) were correctly identified by GPAQ, while 44% (41% – 48%) of the true negatives (i.e., not sufficiently active by accelerometery) were correctly identified by GPAQ (Table 3). In obese participants, (Supplementary Table 1), only 62% (50% – 73%) of true positives were correctly identified by GPAQ. When stratified by site (Supplementary Table 2), GPAQ had a sensitivity of 89% (79% – 95%) and a specificity of 30% (22% – 39%) among Ghanaian participants. In South Africans, the sensitivity was 75% (66% – 82%) and the specificity was 47% (38% – 56%), which was similar to the Jamaican participants, with a sensitivity of 72% (60% – 82%) and a specificity of 50% (42% – 58%). Among the Seychellois participants, the sensitivity was 59% (48% – 70%), while in the US participants, the sensitivity was 87% (70% – 96%). When stratified by sex (Supplementary Table 3), GPAQ had a specificity of 35% (29% – 42%) among the men. Among the women, the sensitivity was 66% (57% – 74%).

**Figure 1:**
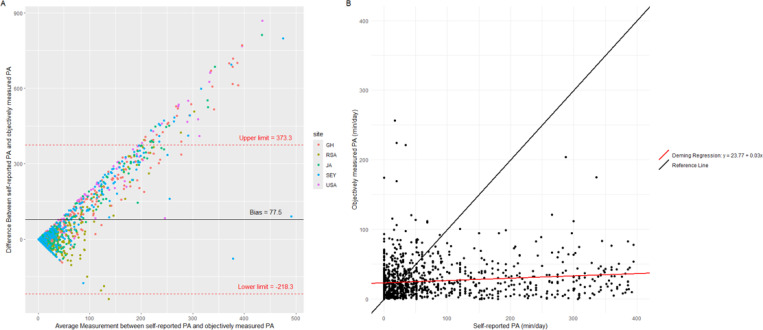
Agreement between objectively measured and self-reported PA (n = 1,161) Panel A: Bland-Altman plot showing the agreement between objectively measured and self-reported PA. The black line represents the bias (the mean difference between objective and self-reported values). The red lines indicate the limits of agreement (bias ±1.96 times the standard deviation of the differences). Each point represents an individual’s difference between objectively measured and self-reported PA, plotted against the average of the two measures. PA: physical activity,y=23.77+0.03x:Deming regression formula.

When calculating the mean PA difference across all five sites using the Bland Altman method, which showed a positive proportional bias, participants self-reported 77 more minutes per day of PA than was objectively measured (Figure 1A). This was confirmed again in Figure 1B where the Lin’s CCC was low (0.04, 95% CI = 0.03 – 0.07) and Deming regression intercept (23.77) and slope (0.03) values that were significantly different from 0 and 1 respectively (p < 0.01).

### Factors associated with correctly self-reporting sufficient PA

The Bland-Altman has a positive bias, and therefore logistic regression analysis was performed among participants who met the PA guidelines according to GPAQ (n=717) to determine factors associated with being sufficiently physically active by accelerometery. Compared to the overall sample (Figure 1), agreement between PA measures was similar in the subgroup of individuals who met PA guidelines according to GPAQ (Supplementary Figure 2). However, the bias, or difference between the two PA measures, is larger in the subgroup compared to the overall cohort (135.2 vs 77.5 respectively).

Among participants who self-reported sufficient PA, compared to the US, participants in each of the other sites had statistically significant higher odds of being sufficiently active according to accelerometry, when holding all other variables constant (Table 4). The greatest difference was seen in South Africa, as participants from South Africa who were sufficiently active by self-reported PA had 5.3 (95% CI = 2.7 – 10.7) times greater adjusted odds of being sufficiently active according to accelerometry, compared to those from the US (p<0.01). In addition, men who self-reported adequate PA had 2.9 (95% CI = 2.0, 4.2) times greater adjusted odds of being sufficiently active according to accelerometry, compared to women who self-reported sufficient PA (p<0.01). In those who were obese, the adjusted odds of being sufficiently active according to accelerometry decreased by 60% (95% CI = 37% – 73%), compared to those who are not obese (p<0.01). Younger age, employment, performing manual labour, having diabetes, and having hypertension were not significantly associated with the outcome.

## Discussion

Our study sought to compare PA, measured by self-report using the GPAQ questionnaire, to PA objectively measured by accelerometery. We found that only 24% of the entire cohort are sufficiently active according to both PA measures. Additionally, 38% of participants self-reported sufficient PA (despite PA being not measured in GPAQ for bouts of PA not lasting for at least 10 minutes consecutively), but do not actually meet the guidelines according to objectively measured PA, as confirmed by the lack of agreement found between the two measures of PA. Men and participants from any site except the US had greater odds of being similarly classified as being sufficiently active with both self-report and device-measured PA, while those with obesity had lower odds of being classified as being sufficiently active using both methods.

These findings may be partly interpreted through the framework of the epidemiologic transition, with fewer participants being sufficiently active in sites further along the epidemiologic transition, such as Seychelles and the US. This may be due to the more sedentary lifestyles. For instance, increased urbanisation and technological advancements may lead people to perceive their daily activities as more active than what they truly are [12], and economic development and urbanisation may result in increased access to recreational facilities, but due to increased sedentary occupations and passive entertainment options, actual utilisation of these facilities may remain low [42]. In addition, strenuous work (e.g. construction) is increasingly carried out by foreign workers in some countries such as the US and Seychelles. An exception to this trend was Ghana, which ranked second highest, after the US, in terms of participants overestimating their PA by GPAQ. This could have been due to site differences in administration of GPAQ. For instance, washing clothes or dishes, which would not be captured through accelerometery, may have been interpreted by participants in some sites as being moderate PA, increasing their self-report PA. However, more research may still be needed to understand the high level of PA over-reporting among the Ghanaian participants in this study.

Our results support previous findings that people are likely to overestimate PA in self-reported measures compared to objective measures [43–47]. Studies conducted in Korea and the US found that self-reported PA was higher than objectively measured PA [44, 46]. In contrast, in a study conducted in France, GPAQ was found to underreport PA according to Bland-Altman, which the authors suggested could be because GPAQ, as opposed to accelerometry, only takes into account PA performed in bouts of 10-minutes or more [48]. Meanwhile, research in African populations is limited. However, in a similar setting to ours in Soweto, South Africa, Watson et al. studied 95 pregnant women and similarly found that GPAQ overestimates MVPA during both the second and third trimester of pregnancy [49].

Our findings show poor agreement between MVPA measured by GPAQ and accelerometery, again supporting previous findings [47, 50, 51]. However, another study in Northern Ireland by Cleland et al. with 101 participants found a moderate, positive correlation between the two measures (r_s_ = 0.48, p = < 0.005) [52]. A study by Chu et al. in Singapore, with 110 participants, investigated the level of agreement between the two measures, but they distinguished between self-administration and interviewer-administered GPAQ. The level of agreement ranged from weak (r_s_ = 0.30 for self-administration) to moderate (r_s_ = 0.46 for interviewer administered) [53]. This contrasts with our findings, as our GPAQ were interviewer-administered, which may have been expected to result in a stronger correlation due to the use of a trained interviewer. However, the weak agreement observed may suggest that even when an interviewer is used, factors such as educational attainment, literacy, numeracy, social desirability bias or misreporting of PA could have negatively impacted the accuracy of the self-reported data [12, 29].

Our regression findings contrast with some previous findings. While we found that men had statistically increased adjusted odds of being correctly classified as being sufficiently active by both PA measures, Wanner et al. found no difference between the sexes for MVPA regardless of the measurement instrument used [47]. The current study shows that obese individuals are statistically less likely to be correctly classified as sufficiently active, while Gorzelitz found that although self-report leads to an overestimation of PA, obesity was not associated with people reporting differently in GPAQ compared to what was found with objectively measured PA [46]. Additionally, Alkahtani reported no statistically significant differenes in MVPA by each measure between obese and normal-weight participants, and that agreement between accelerometry and GPAQ decreases as BMI increases [50]. Future research is needed to better understand these differences in findings.

It is unfortunate that self-reported PA shows less agreement among participants with obesity, as this is a higher-risk group in terms of NCDs, for whom it is particularly important to have accurate PA data for health intervention planning and strengthening. Allocation of resources for PA-promoting interventions, development of the correct interventions for specific groups of individuals such as those with obesity, determining barriers to PA where necessary, and exploring PA as a determinant for health outcomes, may all be misled if PA is not accurately measured. Our findings indicate that although GPAQ is a global questionnaire, it may not be an appropriate measure of PA in all parts of the world. As countries undergo the epidemiologic transition, types of PA, and societal norms surrounding it, change. As such, societal normalisation of a low level of PA could impact self-report [30, 31]. Strategies to improve self-report could include greater cultural adaptation of the constructs of the instrument, such as using show cards, and training of field staff to prevent double counting. Additionally, it would be useful to find a way to capture light PA by self-report.

### Strengths and limitations

To the best of our knowledge, this is the first study to investigate the agreement between GPAQ and accelerometery in populations of African origin from different countries. Another strength of our study is that according to a systematic review conducted by Keating et al., a minimum sample size for questionnaire validation studies should be 90–160 participants for the short version of GPAQ [54]. Our total sample size exceeds this minimum, and even when stratified by site and gender, most groups have sufficient participants, except the Ghanaian and US men. Additionally, our study makes use of Bland-Altman to look at levels of agreement, rather than relying purely on typical correlation measures which assess only the strength of the association between the two measures [48].

A major limitation of the study is that accelerometery is an estimate with many assumptions. Additionally, it doesn’t measure all types of PA, e.g., swimming, weightlifting, and overhead activities [32, 33], admittedly a small source of PA for most people. As mentioned, it doesn’t measure activities which result in limited movement of the centre of body mass, including washing clothes or dishes [45]. Another limitation is that GPAQ asks questions relating to PA performed for 10 or more minutes at a time (while PA for < 10 minutes consecutively is an important source of PA in real life for most people). However, PA is beneficial in intermittent bouts too, as has been highlighted in the 2020 WHO PA guidelines [13, 14, 17], and questionnaires should not be limited by this.

## Conclusion

Our findings underscore the lack of accuracy and agreement of self-reported PA using GPAQ when compared to objectively measured PA using accelerometery, in African-origin populations across different socio-economic settings. Our data suggest that the discrepancy between self-reported and objective measures of PA data tend to differ by settings. These findings may suggest that PA questionnaires may need to be tailored to local settings and calibrated accordingly. Yet, tailoring the questionnaires would raise issues for direct comparison of PA between countries. Given the importance of PA interventions for addressing NCDs, it is crucial to develop methods that ensure valid PA measurement, as this may impact public health strategies and resource allocation. While self-report measures like GPAQ may be useful for large-scale PA surveillance and to identify trends over time, they should be complemented with objective measures to obtain accurate estimates of PA levels. Future research should aim to refine PA assessment tools, considering the diverse needs of different populations. The increasingly wide availability of precise objective PA measurements in simple devices used by many people (smartphones, watches, etc.), some of which have been validated, present interesting new opportunities for collecting reliable PA data. Improving validity and accuracy of PA measurement remains an important challenge that has large implications for addressing the global challenge of physical inactivity and its associated health risks.

## Supplementary Material

Supplement 1

## Data Availability

Data are available upon reasonable request from the corresponding author.

## Abbreviations

BMI: Body mass index
cpm: Counts per minute
CCC: Concordance Correlation Coefficient
CI: Confidence interval
cm: Centimeter
CVD: Cardiovascular disease
GAPPA: Global action plan on physical activity 2018–2030
GPAQ: Global physical activity questionnaire
HDI: Human Development Index
IQR: Interquartile range
kg: kilogram
kg/m^2^: Kilograms per meter squared
mg/dL: Milligrams per decilitre
MET*min/week: metabolic equivalent of task in minutes/week
METS: Modeling the Epidemiologic Transition Study
mmHg: Millimetres of Mercury
MPA: moderate-intensity aerobic PA
MVPA: moderate- and vigorous-intensity PA
NCDs: Non-communicable diseases
NS-SEC: National Statistics Socio-economic Classification
OR: Odds ratio
PA: Physical activity
r_s_: Correlation
SA: South Africa
U.K.: United Kingdom
US: United States
VPA: vigorous-intensity aerobic PA
WHO: World Health Organization
