# Supplementary material for: The utility of physical activity questionnaires among African origin populations; lost in translation?
Source: Res Sq. 2024 Dec 12:rs.3.rs-5529358. Preprint. [Version 1] doi: 10.21203/rs.3.rs-5529358/v1 (PMC11661415; doi:10.21203/rs.3.rs-5529358/v1)
Supplement: Supplement 1 [file NIHPPRS5529358V1-supplement-1.pdf]

Supplementary Files

This is a list of supplementary files associated with this preprint. Click to download.

- UtilityofPAQinMETSIJBNPAsupplementaryinformation.docx
- Tables.docx
